# On the modulation of TRPM channels: Current perspectives and anticancer therapeutic implications

**DOI:** 10.3389/fonc.2022.1065935

**Published:** 2023-02-09

**Authors:** Tania Ciaglia, Vincenzo Vestuto, Alessia Bertamino, Rosario González-Muñiz, Isabel Gómez-Monterrey

**Affiliations:** ^1^ Dipartimento di Farmacia (DIFARMA), Università degli Studi di Salerno, Fisciano, Italy; ^2^ Departamento de Biomiméticos, Instituto de Química Médica, Madrid, Spain; ^3^ Dipartimento di Farmacia, Università degli Studi di Napoli “Federico II”, Naples, Italy

**Keywords:** TRPM channels, modulators, cancer, cell proliferation, autophagy

## Abstract

The transient melastatin receptor potential (TRPM) ion channel subfamily functions as cellular sensors and transducers of critical biological signal pathways by regulating ion homeostasis. Some members of TRPM have been cloned from cancerous tissues, and their abnormal expressions in various solid malignancies have been correlated with cancer cell growth, survival, or death. Recent evidence also highlights the mechanisms underlying the role of TRPMs in tumor epithelial-mesenchymal transition (EMT), autophagy, and cancer metabolic reprogramming. These implications support TRPM channels as potential molecular targets and their modulation as an innovative therapeutic approach against cancer. Here, we discuss the general characteristics of the different TRPMs, focusing on current knowledge about the connection between TRPM channels and critical features of cancer. We also cover TRPM modulators used as pharmaceutical tools in biological trials and an indication of the only clinical trial with a TRPM modulator about cancer. To conclude, the authors describe the prospects for TRPM channels in oncology.

## Introduction

The TRP melastatin (TRPM) channel family comprises eight members (TRPM1-8), representing the largest and most diverse subfamily of the TRP channels. TRPMs are very different in terms of selectivity, activation, and physiological functions. The members of this family share some common characteristics: 1) they are non-selective calcium-permeable cation channels, and almost all family-members conduct Ca^2+^; 2) they are activated by different temperatures, voltages, ion, and lipids such as phosphatidyl inositol(4,5)bisphosphate (PIP2); and 3) some of these channels are organized into cytoskeletal complexes (1–3).

**Figure 1:**
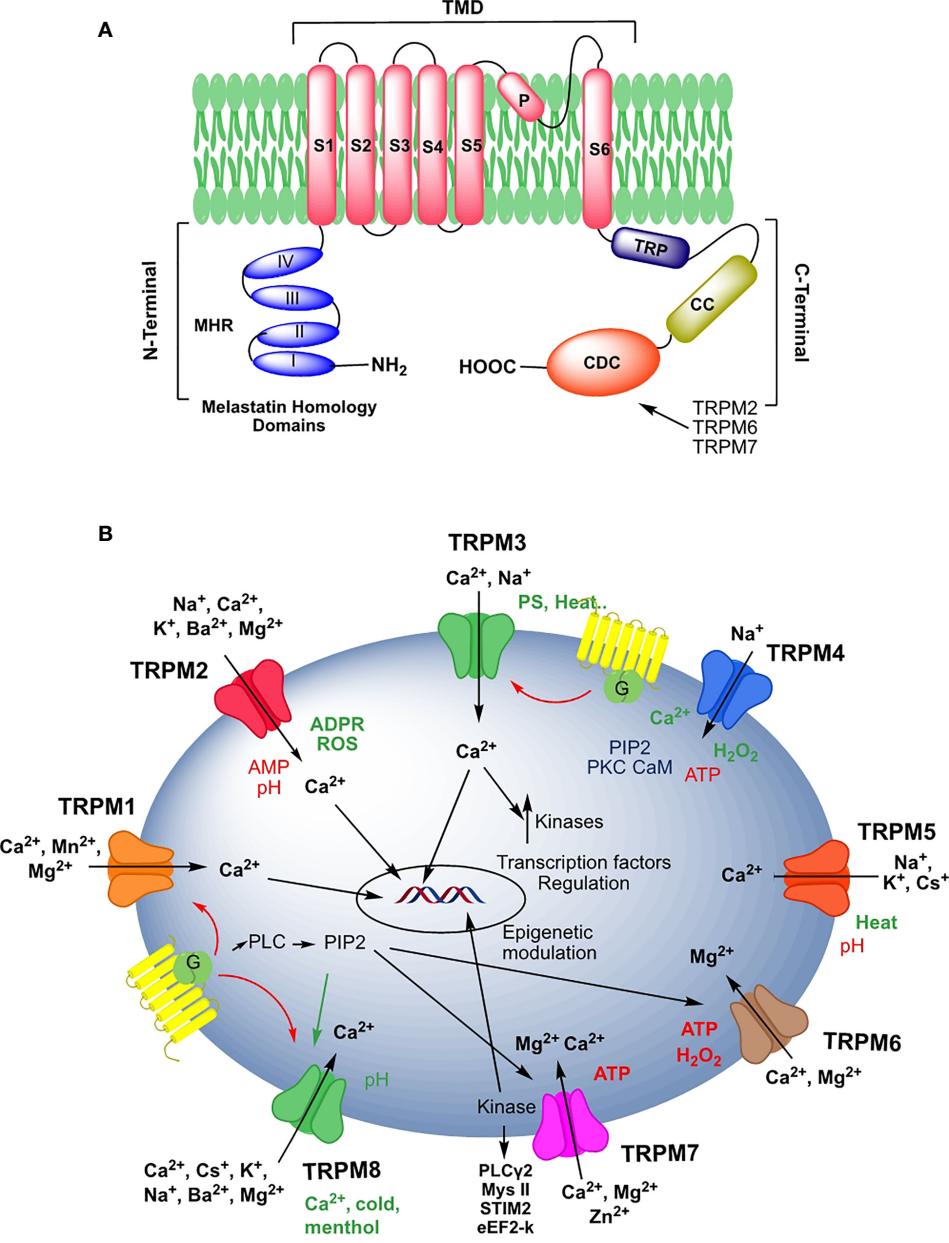
**(A)** Schematic representation of the structural domains of a monomer of the human TRPM family according to the design of Hang et al. (9). **(B)**. Schematic illustrations of ion permeabilities and regulation processes of TRP channels. Color codes, red: channel inactivators; green: channel activators. The designs have been made using Chem Draw Professional 16.0.

TRPM proteins are subdivided based on their structural similarity into four groups: TRPM1/3, TRPM6/7, TRPM4/5, and TRPM2/8 (4) (**Figure 1A**). Through cryo-EM techniques, the complete structures of TRPM2, TRPM4, and TRPM8, and a partial one of TRPM7 have been determined. This allowed approaching the modality of channel assembly, ionic permeation, and modulation binding modes (5–18). Briefly (**Figure 1A**), TRPM proteins contain an N-terminal melastatin homology region domain (MHR), which is involved in channel assembly and external stimuli sensing (9, 13). The transmembrane domain (TMD) comprises six helices with two functional regions, the pore-forming loop between helices S5-S6, and a region in S4-S5 involved in in the activation of these channels. TMD domain is the binding site of Ca^2+^ in three of these channels, TRPM2, TRPM4, and TRPM8, as well as the PIP2 and other pharmacological compounds in TRPM8 (9).

TRPMs’ C-terminal domain varies greatly among members of the subfamily. C-terminal domains contain a highly conserved sequence, the TRP box, implicated in channel anchoring to the plasma membrane, and a coiled-coiled sequence (CC), which contributes to the functional tetrameric assembly (19, 20). In addition, the integrity of these C-terminal domains is required for the cold-induced opening of the TRPM8 channel (21).

After the CC sequence, TRPM2 includes an additional motif, NUDT9-H, which has high homology (50% sequence identity) to the mitochondrial NUDT9 ADPR pyrophosphatase. This region, indispensable for channel function, does not show enzymatic activity functioning as an intracellular ligand-binding site for nucleotides (22, 23). TRPM6 and TRPM7 channels contain a serine/threonine rich α-kinase substrate (S/T) domain and an α-kinase domain (KD) with an interesting enzymatic activity (see below) (24). C-terminal domain of TRPM1/TRPM3, and TRPM4 contains interaction partners for G proteins and ligand-binding sites for nucleotides, respectively (13, 25, 26).

TRPMs are involved in diversified biological processes based on regulating Ca^2+^ signaling, ion homeostasis, interactomes, and/or kinase activity. Many of the TRPMs are involved in cell/organ sensory transduction processes, including the perception of temperature (TRPM2, TRPM3, TRPM4, TRPM5, and TRPM8) and pain (TRPM2, TRPM3, and TRPM8) (27–32), vision (TRPM1) (33), taste (TRPM5) (27, 34), and mechanical forces (TRPM2, TRPM4, TRPM7) (2, 4, 35). Some of them are also involved in ion Mg^2+^ uptake and reabsorption (TRPM6 and TRPM7) (36), and besides modulate secretory processes in various cells all over the body (TRPM3, TRPM2, TRPM3, TRPM4, TRPM5) (31, 37–40). At the same time, mutations or anomalies in the expression and function of these channels could contribute to diverse pathologies, including organ dysfunction, cardiovascular and neurodegenerative disorders, other channelopathies, and cancer (41–47). In the following section, we summarize the functions and characteristics of the channels to proceed to an in-depth analysis of the main pathways modulated by the channels during the initiation and progression of neoplastic diseases.

## TRPM channels: Characteristics and functions

### TRPM1/TRPM3

TRPM1 and TRPM3 share 75% identical amino acids (9). TRPM1 is a non-selective channel, permeable to Ca^2+^, Mn^2+^, and Mg^2+^. TRPM3 shows tissue-specific permeability to mono- and divalent cations related to alternative splicing (48–50).

TRPM1 or melastatin was the first protein of this subfamily to be identified while searching for loci associated with melanoma (51). T*rpm1* maps in chromosome 15 (15q13.3) and encodes five protein isoforms, containing between 1516 and 1643 amino acids, and an intronic microRNA (miR-211) co-expressed with TRPM1 proteins (52). TRPM1 is expressed mainly in melanocytes and in the retina (33, 53). The TRPM1 channel is negatively coupled to mGluR6/Go, through direct interaction with the Gβγ dimer, which, released by Gα(o) dissociation upon mGluR6 activation, closes the channel (25, 54) (**Figure 1B**).

TRPM3 gene, located in chromosome 9 (9q21.12-13), encodes many protein isoforms, most of which have unknown functionality yet, and, analogously to TRPM1, also encodes a microRNA (miR-204) (55–57). TRPM1 and miR-204 are co-expressed in eye cells and pancreatic βcells and are regulated by transcription factors such as Pax6 and MITF (57–59). TRPM3 is also expressed in the kidney, nociceptive neurons, and vasculature muscular layer, as well as in the brain, prostate, ovary, and in sensory bladder afferents, odontoblasts, adipocytes, ciliary body, and oral mucosa (47, 60–63).

TRPM3 is activated by natural ligands, such as sphingosine-1 sulfate, pregnenolone sulfate (PS), nifedipine, the synthetic ligand CIM0216, as well as by noxious heat (29, 31, 40). Combined stimulation by two of these ligands leads to activation of the central pore, which is permeable to Ca^2+^, and an alternative permeation pathway that mediated monovalent cation current and that involves the voltage-sensing domain of TRPM3 (64, 65). 17β-estradiol, progesterone, and its metabolites non-competitively inhibit TRPM3 activation, while dihydrotestosterone behaves as a PS competitive antagonist. TRPM3 activation increases [Ca^2+^]i, which induces activation of different kinases and transcription factors such as ERK, Raf, JNK, CREB, AP-1, Elk-1, and Egr-1 (66–69). Intracellular divalent cations such as cytosolic Ca^2+^ inhibit TRPM1 and TRPM3, whereas Zn^2+^ inhibits TRPM1 but not TRPM3, and Mg^2+^ inhibits TRPM3 activity (47, 70). Analogously to TRPM1, the activation of Gi/o, Gs, or Gq coupled receptors GPCRs inhibits TRPM3 *via* Gβγ liberation (26, 71) (**Figure 1B**).

### TRPM2/TRPM8

TRPM2 are non-voltage-activated channels, porous to monovalent and divalent cations, such as Na^+^, K^+^, Ba^2+^, Ca^2+^, and Mg^2+^. TRPM2 gene is positioned on chromosome 21 (21q22.3) and encodes a 1503 amino acid protein in humans (72). Several alternative splice variants of this protein include TRPM2-S, TRPM2-ΔN, TRPM2-ΔC, TRPM2-SSF, and TRPM2-TE (73–76). TRPM2-S overexpression to suppress endogenous TRPM2 formation and activity has been used to question TRPM2 roles as a regulator of cellular functions mediated by ROS-induced Ca^2+^ signaling (77, 78). TRPM2 is broadly expressed in the brain, including the thalamus, cerebral cortex, hippocampus, striatum, and microglia (79–81). This channel is also detected in the heart, lung, bone marrow, liver, spleen, endometrium, placenta, gastrointestinal tract, and in different pancreatic β-like cells, the salivary gland, endothelial cells, heart, vasculature, and in immune cells (80, 82–84).

H_2_O_2_ and other agents producing reactive oxygen/nitrogen (ROS/RNS) species activate TRPM2 (85). TRPM2 promotes Ca^2+^ influx after activation by ROS and responds to RNS releasing adenosine diphosphate ribose (ADPR) from mitochondria and overproducing TNF-α (85, 86). This activation occurs through the direct binding of the ADPR to the channel’s enzymatic NUDT9-H domain (5, 87, 88). ADRP-induced TRPM2 activation is potentiated by [Ca^2+]^i and arachidonic acid, allowing TRPM2s’ response to changes in intracellular stores-released Ca^2+^ levels, and integrating intracellular signaling events (23, 89). TRPM2 is negatively regulated by adenosine monophosphate (AMP) and cellular acidification (85, 87, 90–93). TRPM2 is inhibited in cells external or internally exposed to pH values of 5-6 (91, 93), while divalent metal cations, Cu^2+^, Hg^2+^, and Zn^2+^ also inhibit TRPM2 by blocking its pore domain (94, 95) (**Figure 1B**).

TRPM8, one of the most studied TRPM channels, was identified during screening of a prostate cDNA library (96). TRPM8 gene is located at 2q37 and harbors genetic diversity with potential functional and phenotypic consequences (97). TRPM8 is a Ca^2+^-permeant but non-selective cation channel (Cs^+^, K^+^, Na^+^, Ba^2+^, Ca^2+^, and Mg^2+^) identified as the physiological sensor of environmental cold (98, 99). TRPM8 is functionally expressed in dorsal root and trigeminal ganglia in skin, teeth, the oral cavity, epithelium, tongue, nasal mucosa, and cornea (100, 101). The channel is also expressed in the colon, lung, liver, kidney, and pancreas, as well as in the male urinary, bladder, genital tracts tissues, and sperm (100, 102, 103). These gender differences in TRPM8 expression in the urinary tract have also been observed in neutrophils expressing the TRPM2 channel, which is lower in neutrophils from older women (104). TRPM8 was also detected in immune system cells, including macrophages (105, 106), bone marrow mesenchymal stem endoplasmic reticulum (ER) membranes (107), and in several areas of mouse brain (108). TRPM8, along with TRPV4, seems also to regulate microglia activities (109).

TRPM8 channels are activated by different stimuli, such as noxious to innocuous (8- 26°C) cold temperatures (28, 98, 99), membrane depolarization (110), increased extracellular osmolarity (111), and in addition by natural and synthetic cooling agents (101). TRPM8 activation/deactivation processes are regulated by post-translational modifications (112, 113), splice variants (97, 114), and modulatory regions (115). Phosphatidylinositol-4,5-bisphosphate (PIP2) changes the voltage-dependent sensitivity of the channel to cold and menthol, acting as a positive endogen TRPM8 modulator (116–118). Nuclear testosterone-androgen receptors (119), interacting proteins (120, 121), and G protein-coupled receptor signaling cascades are also involved in these inhibition/desensitization processes (106, 107). Phospholipase C (PLC) activation and subsequent hydrolysis and depletion of PIP2, or channel phosphorylation by protein kinase C (PKC) are other possible mechanisms of TRPM8 modulation (117, 118, 122, 123). TRPM8 also is inhibited by some GqPCR activation processes through the direct binding of Gαq protein to the channel (116, 124) (**Figure 1B**).

### TRPM4/TRPM5

TRPM4 and TRPM5 are only permeable to monovalent cations and do not conduct Ca^2+^ (125, 126), thus differing from the other TRPM channels. TRPM4 gene is in chromosome 19 (19q13.33), and encodes a 1214 amino acid protein, which works as a voltage-modulated Ca^2+^-activated channel (125, 127, 128). TRPM4 is vastly expressed in the heart, colon, and prostate (125, 128), but also in the central nervous system (129), and in cells of the immunes system (130–132).

Direct activation of TRPM4 by intracellular Ca^2+^ leads to an influx of Na^+^ (133), while ATP, calmodulin, IP3, and protein kinase C-dependent phosphorylation can modify the Ca^2+^-induced TRPM4 activation (134, 135). TRPM4 and TRPM5 gating is also regulated by transmembrane voltage, so that depolarization causes the channel’s activation, whereas hyperpolarization deactivates it (128, 133, 136). H_2_O_2_ induces TRPM4 sustained activation resulting in an increased cell vulnerability to necrotic death (137) (**Figure 1B**).

TRPM5 gene, carried on chromosome 11 (11p15.5), encodes an 1165 amino acids protein (138). TRPM5 is a voltage-modulated Ca^2+^-activated channel that carries monovalent Na^+^, K^+^, and Cs^+^ ions, mediating transient membrane depolarization (127, 136). The TRPM5 channel is expressed in fetal liver, brain, and kidney, and in adult testis, prostate, colon, ovary peripheral blood leukocytes, and taste buds (79, 138). TRPM5 is likewise expressed in insulin-secreting β-cells and in the central nervous system (34, 139, 140).

TRPM5 is physiologically activated by intracellular calcium and heat (136, 141–143). Increasing temperature in the 15-35°C range greatly potentiates TRPM5 inward currents (27). TRPM5 is blocked by extracellular acidification at pH 6.2-5.9 intervals (144). Structurally, residues E830 and H934 in the S5-S6 and S3-S4 linkers are involved in the extracellular pH sensitivity of TRPM5. Interestingly, these residues are not conserved in TRPM4 (144) (**Figure 1B**).

### TRPM6/TRPM7

TRPM6 and TRPM7 exhibit a unique dual functionality within the TRP family, acting as ion channels and kinases (145–147). TRPM6 gene, located on chromosome 9 (9q21.13) codifies for a protein of 2022 amino acids. TRPM6 is permeable to Ca^2+^ and Mg^2+^ and regulated by intracellular levels of Mg^2+^ (148). In this channel, the selectivity filter seems regulated by the amino acid sequence ^1028^GEIDVC^1033^, where the two negative-charged residues D1031 and E1024, are determinants for cation permeation through TRPM6 (149). TRPM6 is expressed in the intestine’s epithelial cells and in the nephron’s distal convoluted tubule (DCT) consistent with its central role in controlling Mg^2+^ homeostasis (79, 124, 148). TRPM6 expression is regulated by hormones such as estrogen, Ang II, and insulin (36, 150–152). Epidermal growth factor (EGF) amplifies the TRPM6 expression and activity, through the signaling pathway ERK/AP-1 (153), while uromodulin increases TRPM6 activity during Mg^2+^ deficiency periods (154). TRPM6-negative endogenous regulators are H_2_O_2_ and ATP-mediated P2X4 receptor signaling (155, 156) (**Figure 1B**).

TRPM7, also named TRP-PLIK, LTRPC7, and ChaK1, was identified by three different groups (145, 157, 158). TRPM7 gene is located on chromosome 15 (15q21.2), encoding an 1863-amino acid protein. TRPM7 is constitutively active and selectively permeable to divalent ions Mg^2+^, Ca^2+^, and traces of Zn^2+^ (157–160). Amino acid residues ^1047^EVY^1049^ in the pore loop provide the TRPM7 selectivity filter (161, 162). Under physiological conditions, TRPM7 inward currents are weak due to substantial downregulation of these channels by high Mg2+ or Mg·ATP concentrations. The decrease in intracellular [Mg^2+^]i, free or bound to ATP, increases the current carried by TRPM7 channels (158, 163). The gating of TRPM7 and TRPM6 is controlled by PIP2 (164, 165). Depletion of PIP2 after activation of PLC-linked GPCRs results in the inactivation of both channels. TRPM6 interacts with PIP2 at the TRP domain through the basic residue R1088, while in TRPM7, the specific contact residues have not been identified yet (164). However, TRPM7 is activated and not inhibited by PLC-coupled GPCR agonists (166). Takezawa et al. reported that GPCR-coupled adenylyl cyclase also enhances TRPM7 activity, and this effect is arbitrated by protein kinase A and cAMP (167).

TRPM7 activity is sensitive to pH (168), polyamines (169), mechanical stretch (170–174), osmotic gradients (175), and chloride and related halides (176). TRPM7 regulates the increase in cytosolic Zn^2+^ efflux induced by increased ROS, as well as blockade of cytosolic Zn^2+^ influx due to decreased glutathione. Oxidative stress perception as well as the release of Zn^2+^ from intracellular storing compartments are also TRPM7-dependent process (160).

TRPM7’s catalytic activity required Mg^2+^ or Mn^2+^ (177). *In vitro*, TRPM7 phosphorylates myosin II isoforms, annexin A1, tropomodulin, eukaryotic elongation factor-2 kinase (eEF2-k), phospholipase C gamma 2 (PLCγ2), and stromal interaction molecule 2 (STIM2) (178–182). TRPM7 kinase auto-phosphorylates threonine and serine residues located in its kinase substrate domain (147, 179, 183, 184). Proteolytic cleavage by caspase results in the release of the kinase domain, which potentiates T cells apoptosis induced by Fas (185). In addition, circulating kinase can translocate to the cell nucleus, where it can phosphorylate histones, thus modulating the chromatin epigenetic landscape, likely in a Zn^2+^-dependent mode (160, 186).

Contingent to the tissue, the structural similarity between TRPM6 and TRPM7 allows the formation of heterotetrametric complexes after the phosphorylation of TRPM7 by TRPM6 (148, 187–189). The TRPM6/7 complex differs from the homomeric TRPM6 and TRPM7 in their permeability to Ni^2+^, conductance, and pore structure. In these channels, the different sensitivity to low pH depends on the difference between the number of negatively charged residues in the pore domain, 7 in TRPM7 and 8 in TRPM6 (36, 157, 162). These assembled channels can modulate the pathophysiological role of both channels (43, 190) (**Figure 1B**).

### TRPM and cancer

**Figure 2:**
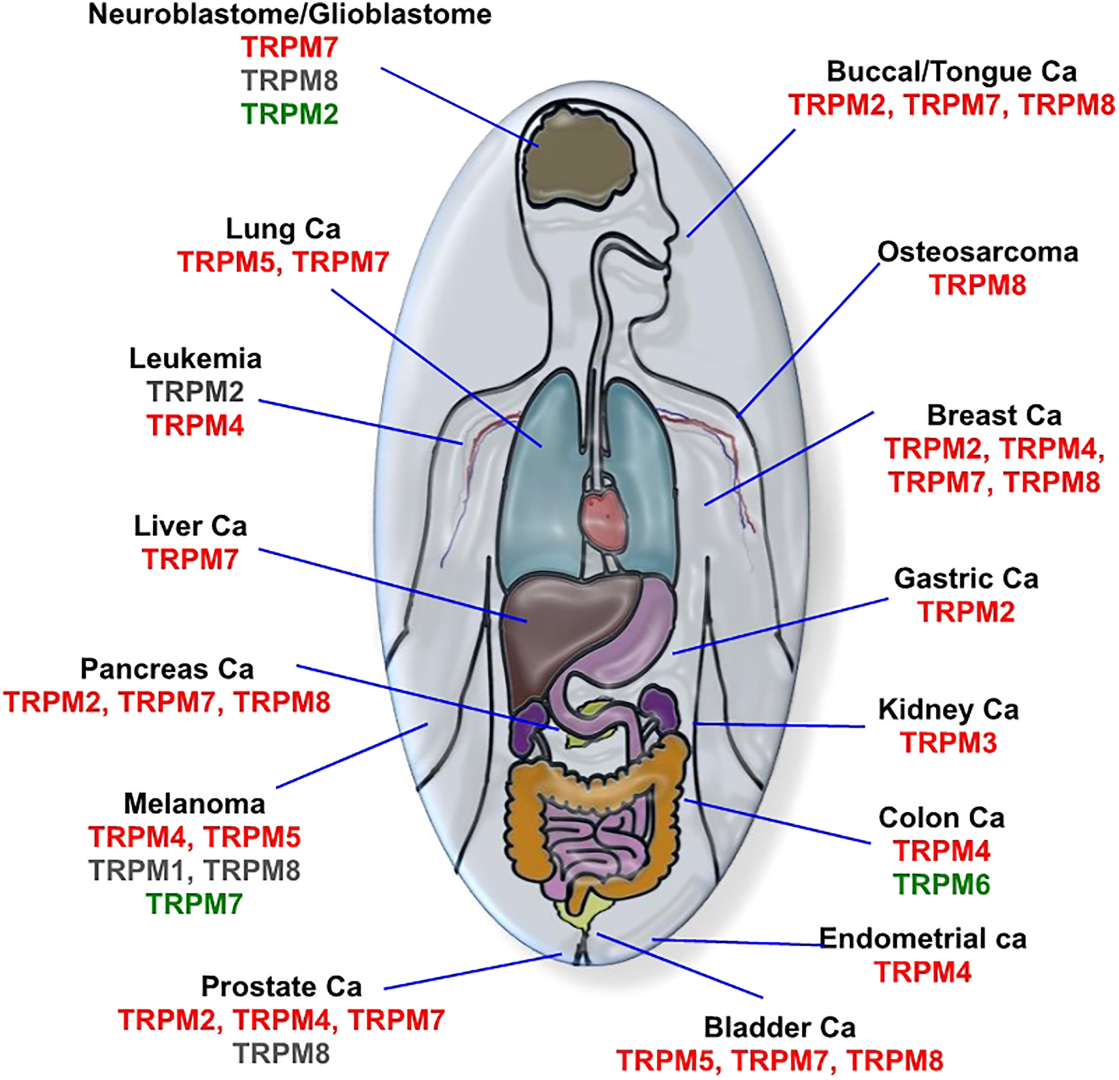
Relation between cancer types and TRPM channel expression. Overexpressed channels with the potential beneficial role of antagonists are indicated in red; down-regulated channels with a possible beneficial effect of agonists are in green; the controversial role of channel according to similar benefits of agonists and antagonists are in grey.

In this section, we discuss recent results involving TRPM channels in oncologic processes, the signaling pathways modulated by these proteins, their mechanisms, and their functions. To facilitate reading, we have summarized in **Figure 2** the cancer types/TRPM channel expression (potential effects of agonists or antagonists) relationships and in **Table 1** the pro/suppressive oncological functions of these channels in certain cell lines, where TRPM modulators have been used as anti-cancer pharmacological tools.

**Table 1:** TRPM functions and their pharmacological modulators in cancer cells.

Channel	Cancer/Cell	Channel Function	Modulators	*In vivo* model	Reference
TRPM2	Breast/MCF-7 and MDA-MB-231	↑ [Ca^2+^]I Promotes integrity DNA	2-APB		Hopkins et al. (191)
	Prostate PC-3	Promote viability Inhibits apoptosis and autophagy	8-Br-ADPR		Tektemur et al. (192)
TRPM3	Renal carcinoma/786-O and A498	↑ [Ca^2+^]I stimulates pro-survival autophagy	Mefenamic acid		Hall et al. (59)
TRPM4	Prostate/DU145	↓SOCE	CBA, NBA, and LBA		Borgström et al. (193)
TRPM5	Lung/ BL6	Promotes migration and metastasis by increased MM9 activity Modulates pHe	Triphenylphosphine oxide (TPPO)	Xenograftnmetastatic melanoma mice model	Maeda et al. (194)
TRPM7	Ovarian cancer/ SKOV3, OVCAR3 HO8910	↑ [Ca^2+^]i Promotes glucose metabolic reprogramming through TRPM7/AMPK/HIF-1α Induces epithelial-mesenchymal transition	Carvacrol	Xenograft ovarian cancer mice	Chen et al. (195)
	Non-small cell lung cancer/ A549 and 95D	Promotes metastasis	Waixenicin A		Liu et al. (196)
	Hepatocellular carcinoma/ HuH6 HuH7	Mg^2+^ influx and phosphorylation of G-protein Prevents replicative senescence	NS8593	Xenograft HCC mice bearing (HuH7)	Mittermeier et al. (197)
	Glioblastoma/ U87, U251 A172	Modulates Ca^2+^ influx Proliferation and invasion Mediates activation of lncHOTAIR	Naltriben Carvacrol	Xenograft GBM mouse models (U87or U251)	Wong et al. (198) Chen et al. (199)
	Bladder/ T24 UMUC3	Promote proliferation, motility	Oridonin Carvacrol	Xenograft model	Che et al. (200) Lee et al. (201)
TRPM8 antagonists and their functions in cancer cells					
TRPM8	Prostate/LNCaP, Cos-7, 22Rv1, C4-2B, PNT-2	Inhibits growth, migration, and invasion of tumor cells in androgen-dependent PCa	2-(5-(benzyloxy)-1H-indol-3-yl)-N-(4-phenoxybenzyl)ethanamine		Di Donato et al. (202)
			(S)-methyl 2-(dibenzylamino)-3-(1H-indol-3-yl)propanoate		
	Prostate/LNCaP, 22Rv1, C4-2B PNT2	Inhibits growth, migration, and invasion of tumor cells in androgen-dependent PCa	Methyl (S)-2-(dibenzylamino)-3-(4-nitrophenyl) propanoate		Di Sarno et al. (203)
			Methyl (S)-2-(dibenzylamino)-3-(3,4-dihydroxyphenyl) propanoate		
			Methyl (S)-3-(4-aminophenyl)-2-(dibenzylamino) propanoate		
	Prostate/LNCaP, 22Rv1	Reduces growth of LNCaP prostate cancer cells	(±)-(R,R)-Bis(1-(4-fluorophenyl)-6,7-dimethoxy-3,4-dihydroisoquinolin-2(1H)-yl)methanone		De Petrocelli et al. (204)
	Prostate/LNCaP, DU145, PNT1A	Anti-proliferative activity	N-(4-tertiarybutylphenyl)-4-(3-chloropyridin-2-yl)tetrahydropyrazine-1(2H)-carbox-amide (BCTC)		Liu et al. (205)
	Squamous carcinoma/HSC3 and HSC4	Augments migration and invasion abilities	RQ-00203078 (RQ)		Okamoto et al. (206)
	Osteosarcoma/U-2 OS, 143B, MG-63, HOS	Promotes growth and metastasis through activation of TGFβ signaling pathway	N-(3-aminopropyl)-2-[(3-methylphenyl) methoxy]-N-(2-thienylmethyl) benzamide (AMTB)	Xenograft tumor of sarcoma cells	Liu et al. (207)
TRPM8 agonists and their functions in cancer cells					
TRPM8	Prostate/LNCaP	Anti-proliferative effects, Inhibits migration	WS-12		Alaimo et al. (208)

### TRPM 1

#### Melanoma

Since its discovery, TRPM1 has been associated with malignant skin pathologies (47, 51). In normal melanocytes, TRPM1 knockdown resulted in reduced [Ca^2+^]i, tyrosinase activity, and intracellular melanin pigment, showing that TRPM1 is involved in Ca^2+^ homeostasis and melanogenesis. Induction of the p53 tumor suppressor by transfection or UVB radiation triggered inhibition of TRPM1 expression and a diminution in both intracellular Ca^2+^ mobilization, and extracellular Ca^2+^ uptake (209). TRPM1 is highly expressed in dysplastic and benign nevi, as well as in cutaneous melanomas, and variably extracted from invasive melanoma, while reduced expression was found in more advanced melanomas (210–212). According to Levi et al., the increase in the miR-211 expression reduces the invasion activity of several cutaneous malignant melanoma cell lines, by decreasing the expression of growth factor receptors IGF2R and TGFBR2, and nuclear factor of activated T-cells 5 (NFAT5) genes (213). Vemurafenib, a BRAF inhibitor, significantly augments the expression of miR-211-5p in tumor-derived extracellular vesicles (EVs) by increasing MIFT expression. Regulation of the TRPM1 gene by MIFT triggers the activation of the survival pathway (214). In melanoma cells, miR-211 transfection decreases sensitivity to vemurafenib, while in a vemurafenib-resistant cell line, inhibiting the mature form of miR-211 (miR-211-5p), diminishes cell prolifer (214). However, in patients with acral melanoma, the increased TRPM1 expression was associated with shorter survival due to tumor progression (215). TRPM1 elevates [Ca^2+^]-cytosolic levels and activates Ca^2+^/calmodulin-dependent protein kinase IIδ (CaMKIIδ), promoting AKT activation after CaMKIIδ/AKT interaction, cell mobility, colony formation, and tumor growth in an *in vivo* (xenograft) melanoma cell model (215).

### TRPM2

TRPM2 has been implicated in several pathological pathways engaging oxidative stress (41, 89, 216–218). After activation of TRPM2 by oxidative stress, the resulting ADPR production can increase [Ca^2+]^i, leading to cell death by enhanced cytokines production, aggravating inflammation and tissue injury (219–223). Other authors suggested that TRPM2-induced Ca^2+^ entry may have a protective role for injured tissues (224, 225).

#### Neuroblastoma and glioblastoma

In neuroblastoma tumor cells and xenograft mice model, TRPM2 modulates both antioxidant response and ROS production, prompting cell survival (78, 216, 226, 227). TRPM2 activation results in the expression of transcription factors and kinases such as HIF-1/2α, CREB, Nrf2, Pyk2, and Src, which contribute to cell survival and proliferation (226–228). TRPM2 inhibition or depletion induces an increase in ROS by mitochondrial dysfunction, while antioxidant diminution results in cell death. Treatment of TRPM2-knockout neuroblastoma cells with doxorubicin decreased cell viability, RNAs encoding for transcription factors, E2F1/2 and FOXM1, and cell cycle regulators, including CDK1, Cyclin B1, CKS1, and PLK (229). Wild-type TRPM2 reestablished the number of living cells, as well as the expression of FOXM1, E2F1, and DNA repair proteins. On the other hand, treatment of DBTRG glioblastoma cells with selenium (Se) and docetaxel (DTX) increases markers involved in ROS production, mitochondrial membrane depolarization, and apoptosis (230). Additive therapy prompted cell death in the glioblastoma cells, *via* TRPM2-mediated increases in oxidative stress and [Ca^2+^]_i_. These effects were reverted after treatment with N-(p-amyl cinnamoyl) anthranilic acid (ACA, **Table 1**), a TRPM2 antagonist.

#### Leukemia

In *in vitro* and *in vivo* models of acute myeloid leukemia (AML), TRPM2 showed a mechanism of action similar to that observed in neuroblastoma. TRPM2 deletion in cells provokes a decrease in mitochondrial potential and calcium uptake, with a reduced antioxidant response (enhanced mitochondrial ROS levels and decreased Nrf2), autophagy inhibition (decreased ULK1, Atg7, and Atg5 protein levels), and bioenergetic modifications (reduced ATF4 and CREB levels). These effects inhibited leukemia proliferation and increased sensitivity to doxorubicin, an effect that was reversed after reconstitution with TRPM2 (231).

In a Trpm2(-/-) mouse model of AML driven by MLL-AF9, the loss of TRPM2 had neither a substantial effect on the progression of leukemic disease nor a synergistic effect with the cytotoxic therapy (232). These divergent results could be attributed to the models used in both experiments.

In Jurkat lymphohematopoietic cancer cells, stably expressing apoptosis-resistant Bcl-2, treatment with *N*-(p-amylcinnamoyl) anthranilic acid (ACA), a TRPM2 inhibitor, followed by irradiation (IR), reduced the CAMKII phosphorylation and hampered the radiation-assisted inactivation of cdc2, which is dependent on the indicated phosphorylation (233). IR stimulated a TRPM2-mediated Ca^2+^-entry that resulted in cell cycle arrest in G2/M, while inhibition of this channel induced cell release from the G2/M phase, promoting cell death (233).

#### Pancreatic cancer

The mutated TRPM2 gene is strongly associated with the survival of patients with pancreatic ductal adenocarcinoma (PDAC), compared to the standard control group. Upregulation of TRPM2 channels could stimulate pancreatic cancer processes (proliferation, migration, and invasion), independently of the tumor cell type (PANC-1, BxPC-3), and of the tumor-bearing mice model. TRPM2 level is significantly increased in PA tissues and negatively correlated to overall survival (234, 235). The action of TRPM2 may be directly due to the activation of PKCα by calcium or indirectly triggered by PKCϵ and PKCδ, through increased DAG, which most likely activates the downstream MAPK/MEK pathway, stimulating cell survival (235).

#### Prostate cancer

TRPM2 is markedly expressed in prostate cancer cells (PCa) compared to the normal prostate epithelium, and its expression fuels in parallel with increasing clinical tumor grade (192, 236). In PC3 cells, H_2_O_2_ addition triggers intracellular Ca^2+^ increase, decreased autophagy marker LC3-II, and induced apoptotic cell death (237). The TRPM2-Ca^2+^-CaMKII cascade is activated by oxidative stress, intensifying the production of intracellular ROS radicals, which are responsible for mitochondrial fragmentation and modification of the mitochondrial membrane potential. The inhibition of early autophagy induction was also observed, managing cell death in TRPM2-expressing tumor cells. Under oxidative stress pressure, TRPM2 knockdown cells shift from cell death to autophagy to help in cell survival (237). Silencing of TRPM2 by siRNA transfection causes a significant increase in autophagic and apoptotic gene expression (ULK1/2, AMBRA1, ATG5/10, BECN1, BAX) at the mRNA level (192). Also, the inhibition of TRPM2 by 8-Br-ADPR led to a significant reduction in the cell viability of PC3 cells (**Table 1**).

#### Breast cancer

TRPM2 was also identified in the foci of human breast cancers (MDA-MB-231 and MCF-7 cells), and inhibition with 2-APB, or silencing through RNAi, reduced tumor cell multiplication (191; **Table 1**). Compared to normal breast cells, TRPM2 inhibition in tumor breast cells produces a significant increase in damaged DNA levels, hypothesizing that TRPM2 activity in the nucleus could facilitate the integrity of genomic DNA, by promoting nuclear calcium influx. In MDA-MB-231 cells, TRPM2 inhibition led to cell death after treatment with doxorubicin or N-methyl-N’-nitro-N-nitrosoguanidine. Silencing TRPM2 selectively increases cell death in both MDA-MB-231 and MCF-7 cells after tamoxifen and doxorubicin treatment, respectively (191, 238). In another appealing work, Gershkovitz et al. demonstrated that the neutrophil cytotoxicity induced by H_2_O_2_ is Ca^2+^- and TRPM2-dependent in several tumor cell lines (239). Silencing TRPM2 in MDA-MB-231 cells showed growth retardation, utter resistance to H_2_O_2_ and neutrophil cytotoxicity, seeding metastatic tumors more efficiently.

#### Gastric cancers and other cancers

The survival of gastric cancer (GS) patients has negatively been correlated to the TRPM2 expression (240). In MKN-45 and AGS cells, TRPM2 knockdown increases apoptosis, and reduces cell growth and mitochondrial metabolism, as denoted by the reduction of both ATP production and mitochondrial oxygen consumption. Concomitantly, a decrease in autophagy and mitophagy-induced proteins (ATG, LC3A/B II, BNIP3) downregulated the c-Jun N- terminal kinase (JNK) pathway, causing accumulation of damaged mitochondria and gastric cancer cell’s death. Downregulation of TRPM2 also sensitized these tumor cells to chemotherapeutic agents, like doxorubicin and paclitaxel (240). These findings are consistent with earlier observations by Wang et al., which demonstrated that the activation of the TRPM2-Ca^2+^-CAMK2 cascade by oxidative stress resulted in phosphorylation of the BECN1 protein, which ultimately inhibited autophagy in liver cells and promotes cell death (241). TRPM2, *via* AKT-mediated epithelial-mesenchymal transition (EMT), also contributes to GC cell invasion and metastasis (242). TRPM2 channel silencing considerably reduced the expression of EMT markers (N- and E-cadherin, twist, and snail), while increasing tumor suppressor PTEN activities. In a mice model, the TRPM2 knockdown eradicated AGS’s tumor proliferation capacity, and produced deregulation of metastatic markers. The same results were obtained in non-small lung cancer (NSCLC) cell lines, A549 and H1299, and in a human lung tumor xenograft SCID mice model (243).

In the tongue carcinoma (SCC) cell line SCC9, it was observed an enhanced expression of TRPM2 located at the nucleus of cancer cells, in contrast with non-malignant human tongue samples. Treatment of SCC9 with H_2_O_2_ for 24 hours induced an increased number of apoptotic cells, while TRPM2 knockdown inhibited SCC9 survival and migration, independently of the apoptotic p53-p21 pathway (244).

### TRPM3

TRPM3 is connected to the control of renal Ca^2+^ ion homeostasis (60), and its expression is upregulated in a type of renal cell carcinoma (ccRCC), with loss mutation of von Hippel-Lindau ligase (VHL, 59). VHL mutation induces a state of pseudohypoxia in the cells due to an increase in the cytoplasmic levels of hypoxia-inducible factor (HIF), which triggers autophagy (245). In human ccRCC cell lines (A498 and 786-O), TRPM3 silencing or treatment with mefenamic acid, a TRPM3 inhibitor, disrupted the formation and growth of tumors (**Table 1**). Mechanistically, Ca^2+^ influx through TRPM3 channels stimulates pro-survival autophagy, with upstream stimulation of Atg-related ULK1 protein, an essential pathway for autophagosome biogenesis, but without mTOR involvement (59). This process was negatively controlled by two VHL-regulated microRNA types (mir-204 and mir-214). VHL loss results in both miR’s lack and autophagy pathway activation (59).

### TRPM4

Reduced expression of the TRPM4 channel drops the proliferation of a cervical cancer-derived HeLa cell line (246). In these cells, TRPM4 suppression stimulated the degradation of β-catenin by GSK-3β, lowering the transcription process dependent on the β-catenin/Tcf/Lef system. Compared to control transfected cells, reduced expression of TRPM4 correlates with a decrease in the number of cells in S phase, and a more significant number of cells in the G1 phase. TRPM4 knockdown reduced the expression levels of survivin and cyclin D1, while TRPM4 overexpression in T-REx 293 cells led to high β-catenin levels and increased cell proliferation (246).

#### Endometrial cancer

TRPM4 has been identified as a protective prognostic gene in endometrial cancer (EC) (247). Low TRPM4 expression in EC patient tissues s was associated with both worse recurrence-free survival and overall survival (248). In AN3CA cells, TRPM4 silencing significantly increases EC progression by up-regulation of mesenchymal markers, N-cadherin, and vimentin. and by decreasing cytokeratin expression, inducing cell proliferation and migration. TRPM4 silencing also leads to reduced p53 and PI3K/AKT/mTOR signaling pathways, strongly implicated in EC pathogenesis.

#### Prostate cancer

TRPM4 channel is among the five candidate driver genes implicated in non-hormonal prostate cancer (PCa) (249). Increased expression of TRPM4 was found in prostate cancer and prostatic intraepithelial neoplasia tissues, compared to non-malignant tissues. In fact, a higher risk of recurrence in PCa patients was associated to this TRPM4 overexpression (249–251). In prostate epithelial (PEC) and prostate cancer (DU145) cells, TRPM4 knockdown significantly increased store-operated Ca^2+^ entry (SOCE). In PC3 and DUA145 androgen-insensitive PCa cell lines, silencing the TRPM4 ion channel decreased cell migration, but not proliferation. Stable CRISPR/Cas9-mediated TRPM4 knockdown DU145 cells showed a rounder shape, lower proliferation, migration, and viability, as well as reduced cell adhesion. However, current TRPM4 inhibitors (CBA, LBA and NBA) did not elicit specific TRPM4 effects in DU145 cells, questioning the function of the ion conductivity of TRPM4 in PCa. In line with the HeLa study (246), decreased TRPM4 levels lead to decreased Akt1 phosphoactivation, probably impaired by an alteration in the EGFR-calcium/calmodulin axis, and by reduced GSK-3β activity. Therefore, the total levels of β-catenin protein are reduced, which, together with a decrease in the transcriptional activity of Tcf/Tcf, induces a diminished proliferation of PC3 cells (252). TRPM4 overexpression in androgen-sensitive LNCaP cells enhances the total levels of β-catenin and the phosphorylation of GSK-3β. Silencing TRPM4 in PC3 cells also decreased migration/invasion ability, *via* a partial reversion of the EMT process, including a decrease in the expression of Snail1, and a substantial change in the MMP9, E-cadherin/N-cadherin, and vimentin expression. Overexpressing TRPM4 in LNCaP cells increases the transcription factor Snail, promoting the repression of E-cadherin and an augment in their migration potential (253).

In PCa tissues, Hong and Yu observed higher and lower TRPM4 and miR-150 expression, respectively, EMT stimulation, as well as β-catenin signaling pathway activation. In PC-3, DU-145, BPH-1, PC-3M-2B4 and LNCaP cells, the upregulation of miR-150 led to the inactivation of the β-catenin signaling pathway (254). Furthermore, either upregulation of miR-150 or knockdown of TRPM4 suppresses proliferation, migration, invasion and EMT *in vitro*, while *in vivo* restrains tumor growth and metastasis (254).

The TRPM4 ion channel is recognized as part of the adhesoma, the protein machinery involved in focal adhesions (FAs) necessary for contractility and migration (255). Blanco et al. propose the interaction of TRPM4 with the microtubule plus-end tracking EB1 and EB2 proteins, which are required for TRPM4 trafficking and functional activity (256). Mutations or inhibition of the TRPM4-EB interaction reduced TRPM4 expression in the plasma membrane and the distribution of channels in ER. In a B16-F10 melanoma model, these mutations diminishes TRPM4-dependent focal adhesion, disassembly rates and cell invasion effects, confirming the TRPM4 channel as an adhesome component (255, 256). The effects of TRPM4 on cell migration are partially mediated by the activation of a GTPase, Rac1. Upon silencing of TRPM4, the serum-induced activation of Rac1 was significantly reduced, diminishing cellular spreading (256).

#### Breast cancer

TRPM4 channel is overexpressed in breast cancer. This expression was associated with EMT and estrogen response gene sets (257), and correlated to negative clinical evolution (257, 258). TRPM4 and K^+^ channel tetramerization domain 5 (KCTD5) protein expressions are increased in different breast cancer samples. KCTD5 positively regulates TRPM4 activity by enhancing its Ca^2+^ sensitivity and promoting cell migration and contractility (258). In addition, KCTD5, as a putative adaptor for the ubiquitin ligase Cullin3-E3, could also promote TRPM4 turnover through ubiquitination (259, 260). In breast cancer stem cells, Verigos et al. hypothesize the involvement of TRPM4 in cell chemoresistance, tumor recurrence, and metastasis. TRPM4 gene was overexpressed in tumorspheres enriched in breast cancer stem cells (bCSCs), and the TRPM4 gene knock-down revealed potential anti-tumor effects by directly reducing stemness properties of bCSCs *in vitro* (261).

#### Colorectal cancer

In colorectal cancer (CRC), the overexpression of TRPM4 has been related to characteristic adverse tumor patterns, such as epithelial-mesenchymal transition and hence infiltrative growth (262). In HCT116 cells, lacking p53 expression, TRPM4 acts as the primary CAN current source conducting large Na^+^ currents. Transient overexpression of p53 reverses this phenotype (263). Silencing TRPM4 in HCT116 resulted in an increased store-operated Ca^2+^ entry as well as in reduced cell viability, proliferation, and invasion, with respect to normal HCT116 cells. Knockout of TRPM4 in the same cells, also induced a shift in the cell cycle towards G1 phase, which seems to be dependent on p53 expression (262, 263). Furthermore, in prostate cells expressing endogenous p53, LNCaPs, a p53 overexpression diminished the currents mediated by TRPM4 channels (263). These authors also observed that the silencing TRPM4-mediated cell cycle switch is abolished in the event of p53 loss, indicating that TRPM4 expression is repressed by p53 (262, 263). TRPM4 regulates Ca^2+^-induced exocytosis in HCT116 cells. which depends on TRPM4 ion conductivity in TRPM4-containing vesicles. Both exocytosis and the delivery of TRPM4 channels to the plasma membrane are mediated by SNARE proteins (264–266). Genome sequencing of affected individuals in different families, non-bearing known CRC predisposing genes mutations, identified variants in the CYBA gene and the TRPM4 gene, leading to a premature stop codon and truncated protein (267). Functional characterization of these variants revealed that TRPM4 knockdown reduced the generation of radical oxygen species in HT-29 and LS174T cell lines, and decreased the production of MUC2 protein, an important component of the intestinal mucus barrier (267).

#### Leukemia

Transcriptome analysis on the impact of azacitidine treatment on four acute myeloid leukemia cell lines identified five up-regulated coding genes, among which TRPM4 is the only surface protein up-regulated (268). In MLL-rearranged leukemia cells, the knockdown of TRPM4 arrested the cell cycle at the G0/G1 phase, impairing tumoral cell growth and proliferation. The authors suggested that this channel could be involved in the regulation of the AKT/GLI1/Cyclin D1 pathway, and that it is behind the pathogenesis of this leukemia (269).

In patients with CD5+ subtypes of diffuse large B-cell lymphoma (DLBCL), the upregulation of TRPM4 mRNA was related to a poorer prognosis compared to CD5− patients (270). The TRPM4 protein was detected in epithelial cells of reactive tonsils, hyperplastic prostates (luminal epithelial cells), kidney distal tubules, and endometrial glands, but was not identified in normal B cells located in lymphoid tissues (271). In activated B cell-like of non-Hodgkin lymphoma DLBCL subtype, the TRPM4 overexpression was linked to reduced overall and progression-free survival (271).

### TRPM5

Single nucleotide polymorphisms (SNP) present in TRPM5 (CG or GG genotype) have been associated with a reduced risk of suffering childhood leukemia compared to the CC genotype (272). In contrast to normal bladder, TRPM5 mRNA is considerably lower in bladder cancer tissues (273). However, TRPM5 protein was not identified in bladder tissues from cancer patients or control subjects. In public-available databanks, the increased TRPM5 mRNA expression was linked to shorter survival in gastric and melanoma cancer patients, but this type of correlation was not found in patients with colorectal, ovarian, breast, or lung cancers (194). TRPM5 protein is highly expressed in BL6 cells, a metastatic B16 melanoma variant. In silencing TRPM5 B16 melanoma cells, these authors observed a reduction of MMP-9 expression, a hallmark of solid tumors associated with EMT, induced by the acidic extracellular pH (194). Mice injected with TRMP5-overexpressed B16-BL6 cells showed an increased degree of acidic pHe-induced MMP-9 expression and lung metastasis. The crucial pHe for MMP-9 induction was not modified by genetic manipulation but merely amplified the inducible MMP-9 percentage, at each pHe (194). In mice, the treatment with triphenylphosphine oxide (TPPO), a known inhibitor of the TRPM5 channel, resulted in a significant reduction of NF-kB activities, lower expression of EMT-associated genes (Vim, Mmp9, Cdh2), and attenuation of spontaneous lung metastasis (194).

### TRPM6

TRPM6 could play oncogenic/tumor suppressive roles through its ability to mediate Mg^2+^ homeostasis and its kinase functions (274, 275).

#### Colon and colorectal cancers

Colon carcinoma LoVo and doxorubicin-resistant LoVo cells showed different cytosolic Mg^2+^ levels. In resistant cells, the total magnesium concentration is higher, but the entry capacity is poorer, than in sensitive cells. In resistant cells, there are decreased TRPM6 and TRPM7 levels due to transcriptional regulation and post-transcriptional events, respectively (276).

In colorectal cancer (CRC), the expression of Mg^2+^ transporters has been investigated in numerous studies (274, 275). TRPM6 mRNA was downregulated in CRC tissues and, therefore, the high expression of TRPM6 channels in CRC patients was correlated to prolonged overall survival (277, 278). These authors also identified hsa-let-7f-1 and hsa-let-7g as the regulatory miRNAs of TRPM6. Pugliese et al. reported that TRPM6 expression was higher in inflammatory (IBD) tumor tissues than in non-IBD CRC, but these facts could not be associated with tumor stage or grade (279). Always in the digestive system, TRPM6 is expressed in the human hepatoma cell lines HepG2 and Huh-7 (280). A study by Pietropaolo et al. hypothesizes that downregulation of TRPM6 contributes to severe hypomagnesemia in cancer patients treated with the monoclonal antibody targeting EGFR, cetuximab (CTX). In human colon carcinoma CaCo-2 cells, CTX reduced the TRPM6-mediated Mg^2+^ influx through interference with the EGF signaling path (281).

### TRPM7

One of the first studies about the implication of TRPM7 in cancer progression informs on the association of an SNP TRPM7 polymorphism (Thr1482Ile) and an increased risk of developing hyperplastic polyps or colorectal adenoma, particularly in patients with a high Ca^2+/^Mg^2+^ intake diet (282). Recent data also identified TRPM7 expression as an indicator of the predisposition to colorectal cancer (CRC) onset and progression in patients with inflammatory bowel disease (279, 283).

#### Prostate cancer

Compared to prostate hyperplasia cells and tissues, the TRPM7 channel is overexpressed in prostate cancer (PCa) cells and tissues and this upregulation has been correlated with poor survival of patients (284). The activation of TRPM7 channels increases the Ca^2+^/Mg^2+^ ratio in serum, promoting cell proliferation in PCa (285). Knockdown of TRPM7 in DU145 and PC3 cells suppressed migration and invasion by reversing the EMT state, thus is, downregulating EMT activators (MMP2 and MMP9) and overexpressing suppressor proteins (E-cadherin) (284). TRPM7 was also required for TGFβ‐induced EMT, which subsequently promoted cell invasion (286). Under hypoxic conditions, TRPM7 knockdown promoted proteasomal HIF-1α degradation and inhibited EMT changes in DU145 and PC3 cells. TRPM7 knockdown increased RACK1 phosphorylation, reinforcing RACK1-HIF-1α interaction, and attenuating the connection between HSP90 and HIF-1α. Deletion of both TRPM7 and HIF-1α suppressed TRPM7-HIF-1α-Annexin-1 signaling, inhibiting hypoxia-induced cell migration and invasion (287).

#### Pancreatic cancer

TRPM7 is essential for pancreatic ductal adenocarcinoma (PDAC) progression and invasion. The over-expression of these channels has been correlated with the increase in tumor size and the advancement of tumor stages, and hence, inversely connected to patient survival (288, 289). In PANC‐1 and MIA PaCa‐2 aggressive PDAC cells, TRPM7 channels mediated the Mg^2+^ entry, which has no effects on cell viability but was a requisite for cell invasion (290). This Mg^2+^ entry prompted heat‐shock protein 90α (Hsp90α) secretion, with the subsequent stabilization of both urokinase plasminogen and pro‐MMP2 activator pathways, stimulating the extracellular matrix degeneration and the PDAC cell invasiveness. In consequence, silencing TRPM7 in PDAC cell lines reduced cancer cell invasion (290). Analogously, in cancer stem (CSCs)-like and metastatic lung cancer cells, TRPM7 silencing induced EMT inhibition, stemness markers, phenotypes suppression, and concomitantly Hsp90α/uPA/MMP2 deregulation (196). TRPM7 channels are implicated in AG-9/VG-6-stimulated MIAPaCa-2 cell migration. Elastin-derived peptides (EDP), AG-9 and VG-6, bind to the ribosomal protein elastin receptor (RPSA), which is overexpressed in human pancreatic tumor tissues, where its interconnection with alpha-6 integrin (ITGA6) regulates the invasion process of PDAC cells (291, 292). This cell migration effect is avoided by TRPM7 channel silencing. EDP treatment did not modify TRPM7 expression but activated the channel without stimulating divalent cation influx into the cytosol. In addition. EDPs treatment induced the co-localization of the TRPM7 channel and the ribosomal protein SA (RBSA). Co-treatment with an RPSA inhibitor, EGCG, and AG-9 prevented the co-localization (292).

#### Breast cancer

In human breast ductal adenocarcinoma (hBDA), the high levels of TRPM7 expression have been interconnected with the Ki67 proliferation index, Scarff-Bloom-Richardson (SBR) cancer grade, and tumor size (293). High TRPM7 mRNA levels have also been linked to lower recurrence‐ and distant metastasis‐free survival. In xenografts, immunodeficient Rag2−/−IL2rg−/− mice, harboring human MDA‐MB‐231 cells, these high TRPM7 levels were required for cancer proliferation and metastasis (294). More recently, it has been described that the increased expression of TRPM7 channels predicts reduced survival in patients suffering from Luminal A breast cancer (295). The authors found that the methylation frequency of the channel has a mean of 42.7% in the whole cohort, while it is much lesser in Luminal A cancer versus other subtypes (33.3% vs. 45.7% in Luminal B, 46.9% in Her2+, and 51.3% in Basal-like). In addition, TRPM7 methylation has been negatively related to metastasis at the lymph node, disease recurrence, and final cancer-induced death. In this type of cancer, de-adhesion of cell-matrix interactions and myosin-II-based cell strains are TRPM7-dependent, and in a murine model of breast cancer, TRPM7 is required for cell metastasis into the lung (296). TRPM7 is also involved in the migration and invasion of MDA-MB-435 cells, in which the regulation of Src and MAPK kinase pathways are dependent on TRPM7 level (297). This suggests that TRPM7’s role is independent of Ca^2+^ entry and involves the channel α-kinase activity, which is required for the phosphorylation of myosin-IIA heavy-chain (179, 296, 297). TRPM7 maintains mesenchymal phenotype in MDA-MB-231 and Hs 578T cells by regulating the EMT transcription factor SOX4 (298). In this respect, the TRPM7 channel reduces the cytoskeletal stress by inhibiting myosin II activity, which mechanistically activates SOX4 expression and, therefore, contributes to metastatic processes of breast cancer cells (298). These results indicate the involvement of TRPM7 channels in both the alteration of mechanical adhesion dynamics and in the cytoskeletal tension reduction, which in the end resulted in increased cell migration (294, 296, 298).

#### Nasopharyngeal carcinoma, ovarian cancer, and neck squamous carcinoma

The TRPM7 role in breast cancer cell migration was also found in other tumors, such as ovarian cancer, and nasopharyngeal and neck squamous carcinomas (195, 299, 300). In ovarian and neck squamous carcinoma samples, the TRPM7 expression was negatively interrelated with the expression of E-cadherin, but positively correlated with N-cadherin, twist, and vimentin expression (195, 228). In ovarian cancer SKOV3 and OVCAR3 cell lines, TRPM7 depletion inhibited migration and invasion, and decreased metastasis to the lung in SKOV3 tumors, therefore prolonging the survival of mice with this type of tumors. Reduction of TRPM7 expression by treatment with MK886, a 5-lipoxygenase inhibitor, or with the intracellular calcium chelator BAPTA-AM, decreased EGF-mediated migration, invasion, and EMT/insulin-like growth factors (IGF), by decreasing the levels of [Ca^2+^]i. TRPM7 silencing also attenuated calcium-related PI3K/AKT activation, which was enhanced by treatment with LY2904002, a PI3K inhibitor (301). The same group proposes TRPM7 as a modulator of metabolic reprogramming pathways in ovarian cancer (195, 302–304). TRPM7 expression levels in SKOV3 and HO8910 cells were positively correlated with glycolysis-related protein (HK2, PDK1) levels, but negatively correlated with oxidative phosphorylation (OXPHOS) pathway-related protein (IDH3B, UQCRC1) levels, suggesting that in ovarian cancer TRPM7 channels promote glycolysis (195). TRPM7 knockout significantly reversed these relationships by shifting glycolysis to OXPHOS. *In vitro* and *in vivo*, silencing TRPM7 increased the activation of AMPK and stimulated the ubiquitination and degradation of HIF-1α, thus attenuating the HIF-1α-enhanced glycolysis, and inhibiting the proliferation of ovarian cancer cells (195). These outcomes may indicate that the glucose metabolic reprogramming in ovarian cancer is regulated by the TRPM7/AMPK/HIF-1α axis (195). TRPM7 silencing not only diminished the glucose uptake, but also ECAR and lactic acid production, while increased ATP, ROS, OCR levels, and NAD^+^/NADH ratios. Consistently, in a xenograft mice ovarian cancer model, the pharmacological inhibition of the TRPM7 activity with carvacrol also decreased the 18F-FDG uptake (195, **Table 1**).

TRPM7 is highly expressed in neck squamous carcinoma (HNSCC) tissues, especially in invasive tissues, and overexpressed in hypopharynx squamous carcinoma (FaDu), tongue squamous carcinoma (SAS), and buccal carcinoma (TW2.6) cell lines, which was associated with poor overall survival rates. The knockdown of TRPM7 negatively regulates the expression of genes and proteins correlated to the calcineurin/NFAT pathway (i.e. NOTCH1, NFATC3) and reduced the percentage of migrating and invasive SAS cells (305). Silencing TRPM7 significantly decreased the E-cadherin/vimentin ratio and suppressed dynamic processes, such as migration, colony and tumorsphere formation, and hence, SAS invasion. These effects involve a negative regulation of the expression of several proteins, like Snail, cyclin D1, c-Myc, SOX2, NANOG, and OCT4. TRPM7-knocked cells alone or treated with cis-platin significantly reduced colony and tumorsphere formation, compared with untreated or cis-platin-treated wild-type SAS cells (305).

#### Bladder cancer

Different works related TRPM7 upregulation to bladder cancer (Bca) cells’ proliferation, motility, and apoptosis (200, 201, 306). TRPM7 knockdown reduced the migration and invasion ability of UMUC3 and T24 cells by suppression of JNK (c-Jun N-terminal kinase), Akt, and Src phosphorylation. Treatment with carvacrol inhibited TRPM7 activity and restricted the tumor size in a xenograft model (201; **Table 1**). Another TRPM7 inhibitor, oridonin, suppressed the proliferative activity, as well as colony-formation and migration capacities of T24 cells, eliciting wide-ranging apoptosis *in vitro*, and delayed tumor growth *in vivo* (200; **Table 1**). On the other hand, treatment with oridonin appreciably improved p53 expression levels, increased caspase-3 activity, and reduced the expression of p-AKT, p-ERK, and TRPM7 channels.

#### Non-small cell lung cancer

In the non-small cell lung cancer (NSCLC) cell line A549, the TRPM7 channel is over-expressed after stimulation of epidermal growth factor (EGF), and an increased cell migration is observed. TRPM7-knockout attenuates the effects of EGF stimulation (307). In TRPM7-rich 95D cells, treatment with Waixenicin A repressed survivin, vimentin, STAT3, uPA, HSP90α, TRPM7, and MMP2 expression levels, while TRPM7-knockout enhanced the anti-CSCs effect above mentioned (196; **Table 1**). Luanpitpong et al. proposed an interesting TRPM7/O-GlcNAcylation regulatory axis as a potential target against lung carcinoma (308). In different cell lines and patient-derived primary cells, the inhibition of TRPM7 and of the enzyme O-GlcNAc transferase (OGT) suppresses the cells’ motility. TRPM7 inhibition also decreases Cav-1 expression, a component of the plasma membrane micro-domains, overexpressed in lung carcinoma, and connected with tumor invasiveness and patients’ poor survival. This inhibition can be reversed by activating O-GlcNAcylation, defining the TRPM7/O-GlcNAc/Cav-1 pathway. Hypo-O-GlcNAcylation of Cav-1, following post-translational TRPM7 inhibition, promotes Cav-1 ubiquitination and subsequent proteasomal degradation, a crosstalk that was also observed with c-Myc. *In vitro* and in experimental lung metastasis *in vivo*, when TRPM7 inhibition repressed O-GlcNAcylation, c-Myc and Cav-1, ubiquitination and then their proteasomal degradation increased, therefore inhibiting cell migration and invasion (308).

Samar et al. also propose the regulatory Ca^2+^influx/O-GlcNAcylation axis, which directly targets ITGA4 and ITGB7 human integrins, as a potential target against the motility and dissemination of myeloma (MM) cells (309). Silencing the TRPM7 channel and, therefore, inhibition of Ca^2+^ entry, efficiently reduced MM cells’ spreading *in vivo*. These results suggest a potential clinical application for both TRPM7 inhibitors and O-GlcNAcylation modulators to treat MM (309).

#### Liver cancer

Inhibition of the TRPM7/myocardin-related transcription factors A and B (MRTFs) axis is another promising strategy to curb hepatocellular carcinoma (HCC) growth (310). The expression of genes implicated in cell proliferation, dissemination, and differentiation is mediated by the serum response factor (SRF), which has MRTFs as coactivators (197). Using HuH7, HuH6, and TRPM7 knockout HAP1 cells, Voringer et al. showed that TRPM7 activation induces RhoA activation, and, successively, the polymerization of F-actin; permits the formation of MRTF-A-Filamin A complex and stimulates MRTF-A/SRF transcriptional activity. The authors hypothesize that the TRPM7 channel activity is necessary to increase Mg^2+^ concentration, required for optimal kinase activity, which is essential for both productive TRPM7-RhoA interaction and MRTF transcriptional activity. Treatment of HuH7 and HuH6 cells with NS8593, a TRPM7 blocker, strongly reduced proliferation by cell cycle arrest in G1 phase. This fact, together with the increase in senescence-associated ß-galactosidase activity, ERK phosphorylation, GTP loading of Ras, and TNFSF10 and p16^INK4a^ expression, indicated that TRPM7 lack of function contributes to oncogene-induced senescence of hepatocarcinoma (HCC) cells. The same results were obtained *in vivo* in the corresponding mice model of HCC xenograft, derived from HuH7 cells (310).

#### Neuroblastoma and glioblastoma

In the human neuroblastoma cell line SHEP-21N, the expression of N-myc oncogene correlated with TRPM7 but not with TRPM6 mRNA expression, probably due to the few numbers of malignant tumors with significant TRPM6 expression (311). N-Myc expression is related to increased cell growth and overexpression of both TRPM6 and TRPM7 channels. N-Myc knockout SHEP-21N cells showed a basal expression of both channels, which was significantly enriched, especially that of TRPM6, by the up-regulation of N-Myc. Analyzing membrane currents, they found that the endogenous TRPM6/TRPM7 currents show decreased Mg·ATP suppression, amplified sensitivity to Mg^2+^, and weak sensitivity to 2-APB inhibition (311). These data support both Ca^2+^ and Mg^2+^ uptake, consistently with the increase of heteromeric TRPM6/TRPM7 channels mediated by N-Myc. Accordingly, the silencing of TRPM6/TRPM7 in these cells induced decreased cell proliferation.

In glioblastoma cells U87, treatment with the TRPM7 activator naltriben induced TRPM7‐like currents through Ca^2+^ influx. It did not alter cells’ viability or proliferation, but increased migration and dissemination (198; **Table 1**). Concomitantly, the author observed improved activation in the MAPK/ERK signaling pathway proteins but not in PI3K/AKT. Treatment of U87 cells with the TRPM7 inhibitor carvacrol decreased cell growth and viability, migration, and dissemination, and induced TRPM7-mediated apoptosis. Carvacrol may regulate dynamic cell processes through the decrease of MMP-2 protein expression and the increased levels of p-cofilin (199).

In xenograft GBM mouse models injected with U87 or U251 cells, the treatment with carvacrol showed a significantly reduced tumor size in both mice, decreased expression of the p-Akt protein, and increased levels of GSK3β (312). TRPM7 expression and activity are not only required for glioma cell proliferation and migration/invasion but also drive glioblastoma stem cells (GSC) plasticity through Notch and STAT3 proliferative activities (313–317). In A172 cells, data from a miRNA microarray analysis revealed down- and up-regulated miRNA whose transcripts are significantly changed after TRPM7 knockdown. There are two TRPM7 mutants with an inactive kinase domain, the Δkinase and the K1648R transfected glioma cells, which have reduced cell invasion, thus indicating the need for an active TRPM7 channel for glioma cell growth, while for cell migration and invasion it seems necessary a functional kinase domain. Overexpression of miR-28-5p suppressed glioma cells’ proliferation and invasion, by upregulating the target Rap1b gene (313). In addition, TRPM7 knockout in A172 glioma cells induced the regulation of a series of lncRNAs (318), of which HOX transcript antisense intergenic RNA (HOTAIR) was the most positively affected by TRPM7 depletion (319). TRPM7-mediated HOTAIR overexpression promoted glioma cell proliferation and invasion. HOTAIR exerted the oncogenic effects partially through TRPM7/HOTAIR/miR301a-3p/FOSL1 axis. TRPM7 mediates the Ca^2+^ influx necessary for NF-κB activation, which transcriptionally activates lncHOTAIR. In turn, lncHOTAIR directly inhibits miR-301a-3p expression, which functionally impedes FOSL1 gene expression activity. As FOSL1 is a key glioma regulator, it is expected that other TRPM7-mediated FOSL1 activations could contribute to glioma pathogenesis, other than HOTAIR and miR-301a-3p.

Differently, Xing et al. showed the mechanisms responsible for autophagy and tumorigenesis process induced by TRPM7 (320). Pharmacological and genetic TRPM7 activation disturbs lysosomes/autophagosome fusion, thus inhibiting the autophagy. TRPM7 activation umpires the releases to the cytosolic media of intracellular Zn^2+^, which abolishes the interaction between VAMP8 and syntaxin 17 (Stx17), two soluble N-ethylmaleimide-sensitive factor-attachment protein receptors (SNARE) located, respectively, in the lysosome and in the autophagosome, and therefore arresting autophagy flux (320). In a panel of cancer and regular cell lines, as well as in xenograft and melanoma lung-metastasis animal models, the autophagy inhibition mediated by TRPM7 activated cell death and blocked cancel cells’ metastasis *in vitro*. *In vivo*, TRPM7 activation limited tumor proliferation and metastasis by inducing ROS production, cell cycle arrest, and apoptosis (321).

### TRPM8

#### Prostate cancer

TRPM8 is overexpressed in prostate tumors compared to non-malignant prostate tissues, and this channel is present in hormone-refractory prostate cancer with higher Gleason grading scores (96, 322, 323). In prostate cells TRPM8 gene expression is dependent on androgen. The androgen receptor (AR), when bound to an androgen (DHT), directly activates the TRPM8 gene promoter (324, 325). Biochemical findings have reported a direct TRPM8 interaction with androgens or their receptors (326). In prostate cancer LNCaP cells, while overexpression of TRPM8 transcript is observed, TRPM8 protein was internalized from its normal plasma membrane localization, ubiquitinated, and degraded *via* proteasomal and lysosomal pathways (327). High internalization and degradation of TRPM8 correlates with greater severity of human prostate cancer cases.

In AR+ prostatic carcinoma cell line LNCaP, silencing TRPM8 or capsazepine blockade of TRPM8 limited cell viability and provoked apoptotic nuclei formation (324). Working with different prostatic tumor cells, Valero et al. reported that TRPM8 knockdown decreased cellular proliferation and arrested cells in the G0/G1 phases, impairing cell cycle progression. The TRPM8 blockers BCTC, AMT, and JNJ41876666 reduce proliferation rates in LNCaP, PC3, and DU145 cell lines, while showing a minimal effect on proliferation in a normal prostate cell line, PNT1A (205, 328). In LNCaP and PC3 cells, TRPM8 depletion inhibited cell proliferation and promoted the chemosensitivity of these cells towards epirubicin, through an increase of p38 and JNK proteins phosphorylation (329). Over-expression of TRPM8 produces anti-proliferative, apoptotic, and anti-migratory effects in PC-3 cells, which are androgen-independent. In these cells, ectopic TRPM8 expression induced G0/G1 cell cycle arrest and expedited starvation-induced cell apoptosis through focal-adhesion kinase inactivation (330). These discrepant effects (activator or suppressor) of TRPM8 on cancer cell growth and survival should depend on the cancer cell types, the molecular phenotype, and the intermediatiors by which TRPM8 channel expression and activity are modulated, without forgetting the contribution of TRPM8 isoforms to the modulation of the whole process (331–333). Treatment of PC3 cells with menthol, a TRPM8 agonist, together with TRPM8 overexpression or AR inhibition, showed an amplified anti-proliferative effect. Another TRPM8 agonist, WS12, when encapsulated into lipid nanocapsules, impaired cancer cell migration ability (334, 335). In a prostate xenograft mouse model, the same treatment limits cell proliferation but also the spread of TRPM8-positive cells to metastatic sites by impairing both the focal adhesion *via* Cdc42, FAK Rac1 and ERK pathway’s inhibition, and the cytoskeleton dynamics (336).

A new TRPM8-controlled anti-invasion mechanism by direct TRPM8/small GTPase Rap1A interaction (PPI) was also described (337). In PC3 cells, the complex formed by TRPM8 and Rap1A, trapped in its GDP-bound inactive form, prevents cell migration and adhesion thus avoiding channel activation at the plasma membrane. Structurally, critical residues for the PPI interaction are Y32 in the sequence of Rap1A and E207 and Y240 in that of TRPM8. These interactions were also found in breast (MCF-7) and cervical (HeLa) cancer cell lines (337).

Alaimo et al. had already shown the positive effects induced by TRPM8 agonists in combination with radio-, hormone- or chemotherapy (208). Extracellular and intracellular stimuli, if are prolonged over time and produce minimal increases in [Ca2+]i, may be behind of the damaging cellular stress. In LNCaP_FGC_ cells overexpressing the channel, treatment with WS-12 (48 h) in combination with either enzalutamide or docetaxel increases cell death percentage, from about 20% after single treatments to nearly 60% (208). We also found that tryptophane-derived TRPM8 agonists were able to show anti-PC potential in LNCaP cells, despite antagonists appeared more potent and reliable compounds in the same model (202).

Petrocelli et al. identified a powerful and selective tetrahydroisoquinoline-based TRPM8 antagonist, with strong antiproliferative activity in LNCaP prostate cancer cells (204). Analogously, tryptophane-derived TRPM8 antagonists inhibit proliferation in the LNCaP cell line as well as in metastatic and resistant tumor cell C4-2B, 22Rv1, and DU-CaP lines. In AR+ LNCaP, selected TRPM8 modulators mitigated migration and invasiveness (202). These effects are maintained in both LNCaP spheroids expressing AR and the castration-resistant prostate cancer (CRPC) model. This model is usually resistant to deprivation of androgen therapy (ADT) and is considered the most aggressive form of PCa. No effects were detected in PCa cells devoid of the AR receptor (202, 203). These TRPM8 antagonists interfere with AR/TRPM8 crosstalk by a non-genomic mechanism abolishing the AR/TRPM8 complex assembly, and counteracting the increase in intracellular Ca^2+^ levels.

To our knowledge, only a modulator of TRPM8 (D3263, Dendreon Corporation) reached a clinical trial (phase I) for the management of advanced solid tumors (NCT00839631, 101). D3263 is an orally active, TRPM8 agonist able to slow down tumor progression in advanced prostate cancer patients and in benign prostatic hyperplasia. No results have been published to date about this clinical study.

#### Pancreatic cancers

Pancreatic adenocarcinoma (PC) cell lines overexpressed TRPM8 channels, and this fact has been correlated to advanced TNM, vast tumor size, and distant metastasis. Patients having high TRPM8 expression show worse DFS and OS than patients with low TRPM8 levels (323, 338). In pancreatic adenocarcinoma PANC-1 and BxPC-3 cell lines, silencing TRPM8 reduced cell proliferation and showed impaired cell cycle progression, causing cells to arrest in the G1 phase and, hence, decreasing the number of cells entering S phase (339). Consistently, it was observed an increase of p21^CDKN2A^ and p27^CDKN2B^ cyclin-dependent kinases. However, an increase in the proportion of apoptotic cells compared to control cells was not observed, but they exhibited features of replicative senescence by inducing senescence-associated β-galactosidase (SAβG) expression. These findings indicate that the TRPM8 channel is essential for sustaining uncontrolled cancer cell growth, by regulating cell cycle phases and replicative senescence (288). TRPM8 regulation by LCK, a crucial lymphocyte-specific tyrosine kinase in regulating T-cell functions, reveals that the phosphorylation at Y1022 of the TRPM8 protein is important for pancreatic tumor cell proliferation, migration, and tumorigenesis (340). The phosphorylation process, as well as the 14-3-3ζ/TRPM8 interaction, regulates TRPM8 multi-merization and, therefore, increased current density. LCK significantly enhanced this interaction, whereas mutant TRPM8-Y1022F and knockdown of the 14-3-3ζ reduced LCK-induced TRPM8 oligomerization. In addition, the phosphorylation of TRPM8 at Y1022 inhibited, in turn, LCK Tyr505 phosphorylation, thus modulating LCK ubiquitination and activity. In AsPC-1 and PANC-1 cells stably expressing WT-TRPM8, control vector, or TRPM8-Y1022F mutant, it was observed that WT-TRPM8 considerably increased tumor cell proliferation and showed a significantly higher migration capacity in PANC-1, compared to control vector-containing cells. Diversely, the TRPM8-Y1022F mutant impaired cell proliferation and migration processes in this cell line. *In vivo* assays in a mice PANC-1 cell-derived xenograft model of pancreatic tumors, expressing the control vector, WT, and Y1022F-TRPM8, showed up-regulated TRPM8 mRNA expression in tumors expressing WT or Y1022F. Histopathologic analyses indicated a significant increase in tumor volumes and weights, as well as in the Ki67 antigen expression, in WT-TRPM8 tumor tissues compared to controls. In mutant TRPM8-Y1022F tissues, a smaller increase in volume, weight, and Ki67 antigen expression was observed compared to WT-TRPM8 tumors (340).

#### Breast cancer

In ER+ grade I breast adenocarcinomas, TRPM8 expression is normally upregulated (293, 341), with comparatively higher expression of TRPM8 mRNA in the highly invasive MDA-MB-231 cell line. In MDA-MB-231 cells, TRPM8 knockout reduces migration and invasion, decreasing the EMT-related markers (GSK-3ß phosphorylation, Snail, and Akt). Contrastingly, in the low-aggressive MCF-7 cell line, TRPM8 overexpression enhances invasion and migration, stimulating EMTs (293). TRPM8 activity might be hormone-dependent in these cells, and its expression is regulated by estrogen receptor alpha (ERα) and estrogens, and correlated with the tumors’ estrogen receptor status (293, 341, 342). TRPM8 promotes metastasis in MDA-MB-231 or MCF-7 cells by regulating epithelial-mesenchymal transition (EMT) *via* AKT/GSK-3β pathway activation (342, 343). However, it has also been described that TRPM8 was not expressed in MDA-MB-231 cells and that the TRPM8 transcript is absent in half of the studied breast cancer cell lines, emphasizing that the relevance of TRPM8 as a therapeutic target is very limited in this case (344).

#### Glioblastoma cancer

TRPM8 affects glioblastoma (GBM) cell migration rate by stimulation with specific agonists, such as menthol and icilin, which mediated a substantial increase in [Ca^2+^]i (345, 346), while contributes to proliferation, survival, and local tumor invasion in the case of U251 glioblastoma cells (347). In T98G and U-87MG cell lines, the activation of TRPM8 channels by icilin produced an important increase in migration speed and chemotaxis, Consistently, TRPM8 downregulation by RNAi or through a specific channel blocker, BCTC, decreases both cell migration and transfer chemotaxis (346, **Table 1**). The stimulation of large-conductance Ca^2+^-activated K^+^ ion channels (BK channels), could be a possible mechanism behind the cell migration in glioma, induced by TRPM8-mediated Ca^2+^ influx (345). TRPM8 activation by agonists increased the probable opening of single BK channels (345, 346), while TRPM8 activation and upregulation by ionizing radiation increased the Ca^2+^ influx (346). In human glioblastoma cells, the TRPM8 channel stimulated cell progression to the S phase, and mitosis, inducting cyclin-dependent kinase CDC2, Ca^2+^/calmodulin-dependent protein kinase II (CaMKII) isoforms, and phosphatase CDC25C (346). In contrast, TRPM8 knockdown or inhibition impaired cell cycle progression, DNA repair, and clonogenic survival, while inducing apoptotic cell death (346).

#### Osteosarcoma

The proliferative role of TRPM8 in osteosarcoma (OSS) is demonstrated in osteosarcoma cancer cells lines, MG-63 and U2OS, where TRPM8 is aberrantly over-expressed (348). TRPM8 knockdown in these cells impaired regulation of the [Ca^2+^]i, and decreased cyclinD1, Cdk4 expression, therefore blocking cell cycle in G0/G1, and also p-GSK-3β and p-Akt expression, inhibiting metastasis. In silencing TRPM8 cells treated with epirubicin, the inhibition of Akt-GSK-3β pathway with suppression of FAK and p44/p42 phosphorylation, amplified epirubicin-induced cell apoptosis (349). Overexpression of TRPM8 transcript and protein is concomitant to higher clinical stages, distant metastasis, and feebler disease-free survival (350).

Treatment of several osteosarcoma cell lines (143B, U2OS, HOS, and MG-63), with AMTB, a TRPM8 antagonist, results in suppressed proliferation and metastasis, and induces cellular apoptosis (207). In 143B and U2OS, incubation with AMTB for 24 h increases apoptosis rate, raising both poly (ADP-ribose) polymerase (PARP) and cleaved caspase-3 levels (207). In nude mice xenograft model, AMTB treatment augmented tumor cells’ sensitivity to cisplatin, by smodering Smad2 and Smad3 phosphorylation and, therefore, repressing the activation of TGFβ signaling, which was implicated in tumorigenesis and tumor progression (207, 351).

#### Squamous cell carcinoma

TRPM8 is overexpressed at the plasma membrane and in the intracellular region of oral squamous cell carcinoma (SCC), promoting the proliferation of these cancer cells. RQ-00203078, a potent and selective antagonist of TRPM8, strongly inhibits migration and invasion capability of HSC3 and HSC4 SCC lines, markedly reducing clonogenic potential and calcium entry (206; **Table 1**).

Recently, Huang et al. observed that the overexpression of TRPM8 channels increased basal autophagy levels, while TRPM8 knockdown reduced it, in different types of mammalian cancer cells, including cervical cancer (HeLa), breast cancer (MDA-MB-231 and MCF7), embryonic kidney (HEK293) and colorectal carcinoma (HCT116) (9). The mechanism of TRPM8 autophagy regulation involves the activation of autophagy-associated signaling proteins, AMPK and ULK1, and phagophore formation. In breast cancer cell lines, the authors hypothesized the formation through the cytoplasmic C-terminus of TRPM8 of a TRPM8-AMPK complex protein, which stimulates AMPK phosphorylation and activation, and subsequent the ULK1 activation to enhance basal autophagy (352).

### Perspective

During the last decade, accumulative research indicates the close relationship between the cancer hallmarks and the deregulation of one or more ion channels. TRPM proteins are part of that family that plays a crucial role in the mechanisms of cancer cell proliferation, invasion, and survival. However, forthcoming treatments with TRPM modulators still need more comprehensive and coordinated basic and applied investigations.

We think that the recently disclosed structural data, and the new structures that undoubtedly will come soon, will aid in a more rational design of modulators. As proof, we refer to the channels with more information on structural data, including right now an open channel state (353), and a more significant number of modulators: TRPM8. This information will also be valuable in the case of TRPM6 and TRPM7 due to the complexity of their interactions and the biological effects derived.

TRPM channels may represent key support for the success of more conventional anticancer strategies such as chemotherapy and radiotherapy. By damaging target cells, these therapies trigger many stress responses, such as ion channel activation. Stimulation of sensitizing channels (TRPM2 or TRPM8) can intensify insults provoked by these therapies, whereas modulation of other channels can circumvent intrinsic chemo(radio)resistance.

Since the TRPM8 receptor is closely related to the control of core body temperature, the possibility of oral or systemic treatments based on TRPM8 antagonists is minimal, as it has been shown with compounds that have reached, but not passed, early clinical trials (354). However, TRP modulators in topical preparations can be an alternative solution. Two cosmetic preparations have recently been marketed to help in restoring the balance of nociceptors in chemotherapy-induced peripheral neuropathy, related to the overexpression of TRPM8 channels (Alodia creams, https://atikapharma.com/es/products/alodia-manos). The preparation of TRPM-based biosensors for cancer diagnosis and the development of tumor-targeted nanomedicines containing TRPM modulators, alone or in combination with known chemotherapeutic agents, could also be contemplated soon in this field.

As described in this review, several miRNA/TRP channel pairs seem to play a key role in tumor biology such as TRPM1, TRPM3, and TRPM4. New therapies based on siRNA and antisense oligonucleotides should be considered in the search for specific anti-tumor targets and modulators centered on TRPM channels (317).

Improved knowledge of the TRPM/receptors interactome as well as on the functional association between different ion channels, is another fundamental aspect to discover cellular pathways operating in TRPM-associated cancers. The search for synergy between channels/receptors/proteins or between different channels involved in the regulation of the same or complementary cancer signaling pathways can be the basis for the design of more effective TRPM modulators.

Another issue is finding the most suitable animal model to validate TRPMs as potential targets and translate efficacy and safety discoveries to human studies. It is commonly accepted that integrating relevant data from humans at the early stages of the drug discovery process strengthens the probability of success (355, 356). In this regard, the recent availability of multi-omics epidemiological tools and large-scale clinical datasets will allow a more reliable prediction of concerns related to the direct modulation of a particular target in humans (357). The efficacy and safety signal results from these “virtual” trials are likely to drive discussions about the benefits and risks for each individual TRPM channel.

In summary, the functional modulation of TRPM channels will provide promising opportunities for new antitumor agents’ development, and behind cancer, for peripheral neuropathies and other disabling diseases.

## Author contributions

All authors have read and agreed to the published version of the manuscript.

## Glossary

**Table:**

CSCs	cancer stem cells
cryo-EM	cryogenic electron microscopy
TMD	transmembrane domain
NUDT9	Nudix hydrolase 9
ADPR	Adenosine diphosphate ribosyl
AMP	adenosine monophosphate
PLIK	phospholipase C-interacting kinase
PAX6	paired box 6
MITF	melanocyte inducing transcription factor
Raf	rapidly accelerated fibrosarcoma
ERK	extracellular signal-regulated kinase
JNK	c-Jun N-terminal kinases
AP-1	Activator protein 1
CREB	cAMP response element-binding protein
EGR-1	early growth response protein 1
ELK1	ETS transcription factor
P2X4	purinergic P2X4
eEF2-k	eukaryotic elongation factor-2 kinase
PLCγ2	phospholipase C gamma 2
STIM2	stromal interaction molecule 2
IGF2R	insulin like growth factor 2 receptor
TGFBR2	transforming growth factor beta receptor 2
NFAT5	nuclear factor of activated T cells 5
BRAF	v-raf murine sarcoma viral oncogene homolog B1
AKT	protein kinase B
HIF	hypoxia-inducible factor
FOXO3a	forkhead box protein O3
Nrf2	nuclear factor erythroid 2–related factor 2
FOXM1	forkhead box M1
E2F1/2	E2F transcription factor ½
CDK1	cyclin dependent kinase 1
PLK1	Polo like kinase 1
CKS1	Cyclin-dependent protein kinase regulatory subunit 1
DBTRG	Denver brain tumor research group
ULK1	Unc-51 like autophagy activating kinase 1
ATG7	autophagy related 7
ATG5	autophagy related 5
ATF4	activating transcription factor 4
MLL	mixed-lineage leukemia 1
CaMKII	Calcium/calmodulin-dependent protein kinase II
PANC-1	pancreatic cancer cells
PKC	protein kinase C
MAPK	mitogen-activated protein kinase
MEK	mitogen-activated ERK kinase
LC3-II	microtubule-associated protein 1A/1B-light chain 3 tipe II
ULK1	Unc-51 like autophagy activating kinase 1
AMBRA1	autophagy and Beclin 1 regulator 1
ATG10	autophagy related 10
BECN1	beclin 1
BAX	BCL2 associated X
MDA-MB-231	M.D. Anderson-Metastatic Breast 231
AGS	adenocarcinoma gastric cell line
MKN45	human gastric adenocarcinoma
BNIP3	BCL2 interacting protein 3
LC3A	microtubule-associated protein 1 light chain 3
PTEN	Phosphatase and tensin homolog
NOD	non-obese diabetic
SCID	severe combined immunodeficiency
ccRCC	clear cell renal cell carcinoma
IGF	insulin-like growth factor
EGFR	epithelial growth factor receptor
mTOR	mechanistic target of rapamycin
GSK	glycogen synthase kinase-3
T-REx 293	tetracycline repressor protein
AN3CA	metastatic undifferentiated EC
EMT	epithelial-mesenchymal transition
PI3K	phosphoinositide 3-kinase
CRISPR	clustered regularly interspaced short palindromic repeats
CBA	4-chloro-2-[2-(2-chloro-phenoxy)-acetylamino]-benzoic acid
NBA	4-chloro-2-(2-(naphthalene-1-yloxy) acetamido) benzoic acid
LBA	4-chloro-2-(2-(4-chloro-2-methylphenoxy)propanamido) benzoic acid)
TCF/LEF	T cell factor/lymphoid enhancer factor
AKT1	AKT serine/threonine kinase 1
SNAI1	snail family transcriptional repressor 1
MIR150	MicroRNA 150
Rac1	Ras-related C3 botulinum toxin substrate 1
SNARE	N-ethylmaleimide-sensitive factor-attachment protein receptor
CYBA	Cytochrome B-245 alpha chain
MUC2	Mucin 2, oligomeric mucus/gel-forming
GLI1	GLI family zinc finger 1
MMP-9	matrix metallopeptidase 9
NF-κB	nuclear factor kappa-light-chain-enhancer of activated B cells
MMP-2	matrix metalloproteinase-2
TGFβ	transforming growth factor β;’RACK1, receptor of activated protein C kinase 1
uPA	Urokinase-type plasminogen activator
EGCG	Epigallocatechin-3-gallate
RAG-2	recombination activating gene 2 protein
IL2RG	interleukin 2 receptor subunit gamma
SOX4	SRY-Box transcription factor 4
BAPTA-AM	1,2-bis(2-aminophenoxy)ethane-N,N,N′,N′-tetraacetic acid tetrakis(acetoxymethyl ester)
HK2	Hexokinase 2
PDK1	phosphoinositide-dependent kinase-1
IDH3B	isocitrate dehydrogenase NAD(+) 3 non-catalytic subunit beta
UQCRC1	Ubiquinol-Cytochrome C reductase core protein 1
ECAR	extracellular acidification rate
OCR	oxygen consumption rate
18F-FDG	fluorodeoxyglucose F 18
NFAT	nuclear factor of activated T-cells
NFATC	nuclear factor of activated T cells 3
Notch 1	Notch homolog 1 translocation-associated
c-myc	cellular myelocytomatosis oncogene
SOX2	SRY-Box transcription factor 2
Oct-4	octamer-binding transcription factor 4
STAT3	signal transducer and activator of transcription 3
CAV1	Caveolin 1
ITGA4	Integrin subunit alpha 4
ITGB7	integrin subunit beta 7
2-APB	2-aminoethyl diphenyl borinate
GBM	glioblastoma
BCTC	4-(3-Chloro-2-pyridinyl)-N-[4-(1,1-dimethylethyl)phenyl]-1-piperazinecarboxamide
AMTB	N-(3-Aminopropyl)-2-[(3-methylphenyl)methoxy]-N-(2-thienylmethyl)benzamide
Cdc42	cell division cycle 42 protein
FAK	focal adhesion kinase
Rap-1A	Ras-related protein 1
BK	big potassium
Cdk4	Cyclin-dependent kinase 4
TNM	tumour, node, metastasis (staging cancer).
